# Greenhouse gas and ammonia emissions from stored manure from beef cattle supplemented 3-nitrooxypropanol and monensin to reduce enteric methane emissions

**DOI:** 10.1038/s41598-020-75236-w

**Published:** 2020-11-09

**Authors:** Jennifer L. Owens, Ben W. Thomas, Jessica L. Stoeckli, Karen A. Beauchemin, Tim A. McAllister, Francis J. Larney, Xiying Hao

**Affiliations:** 1 Agriculture and Agri-Food Canada, Lethbridge Research and Development Centre, 5403 1st Ave S., Lethbridge, AB T1J 4B1 Canada; 2 Agriculture and Agri-Food Canada, Agassiz Research and Development Centre, 6947 Lougheed Hwy, Agassiz, BC V0M 1A0 Canada

**Keywords:** Biogeochemistry, Environmental sciences

## Abstract

The investigative material 3-nitrooxypropanol (3-NOP) can reduce enteric methane emissions from beef cattle. North American beef cattle are often supplemented the drug monensin to improve feed digestibility. Residual and confounding effects of these additives on manure greenhouse gas (GHG) emissions are unknown. This research tested whether manure carbon and nitrogen, and GHG and ammonia emissions, differed from cattle fed a typical finishing diet and 3-NOP [125–200 mg kg^−1^ dry matter (DM) feed], or both 3-NOP (125–200 mg kg^−1^ DM) and monensin (33 mg kg^−1^ DM) together, compared to a control (no supplements) when manure was stockpiled or composted for 202 days. Consistent with other studies, cumulative GHGs (except nitrous oxide) and ammonia emissions were higher from composted compared to stockpiled manure (all *P* < 0.01). Dry matter, total carbon and total nitrogen mass balance estimates, and cumulative GHG and ammonia emissions, from stored manure were not affected by 3-NOP or monensin. During the current experiment, supplementing beef cattle with 3-NOP did not significantly affect manure GHG or NH_3_ emissions during storage under the tested management conditions, suggesting supplementing cattle with 3-NOP does not have residual effects on manure decomposition as estimated using total carbon and nitrogen losses and GHG emissions.

## Introduction

Agricultural activities represent roughly one third of global anthropogenic greenhouse gas (GHG) emissions^1^. Cattle are responsible for up to 17% of global methane (CH_4_) production, most of which is from ruminant enteric fermentation^2^. Methane from enteric fermentation and manure in beef cattle production represents between 40 and 70% of the total GHGs from North American beef operations^3,4^, and animal housing and manure storage accounts for between 5 and 15% of total nitrous oxide (N_2_O) emissions^5^.

In North America, finishing beef cattle are fed mixed diets in commercial feedlots where they regularly receive additives. The ionophore monensin (Rumensin, Elanco Animal Health, Guelph, Ontario, Canada) is one of the most common additives administered to cattle as it improves feed efficiency^6^ and reduces the risk of bloat and rumen acidosis^7,8^. Ionophores like monensin may also reduce enteric CH_4_ production by decreasing the availability of primary substrates (hydrogen and formate) for ruminant methanogens^8,9^. In some cases, dietary supplementation of monensin has been found to alter stored manure chemical composition^10^ and may lower CH_4_ emissions from manure^11^, but the response is influenced by cattle diet, age and breed^12^.

3-Nitrooxypropanol (3-NOP, DSM Nutritional Products AG; Kaiseraugst, Switzerland) is an investigative material that has been developed to reduce enteric CH_4_ production. 3-Nitrooxypropanol works by specifically inhibiting the methyl-coenzyme M reductase, an enzyme in methanogenic archaea that catalyses the CH_4_-forming reaction in the rumen^13^. The inhibitory effects of 3-NOP in the rumen are transient^13–15^ as 3-NOP is degraded in the rumen by its own mode of action and therefore it is highly unlikely to reach the lower digestive tract to be excreted in manure^13^. Past beef cattle studies have reported that 3-NOP does not significantly affect the digestibility of organic matter, crude protein, neutral detergent fibre, acid detergent fibre or starch^16^. However, it is unknown whether 3-NOP supplementation alters manure chemical properties through its effects on rumen fermentation in such a way that it will affect manure decomposition during storage.

Storage affects manure decomposition and therefore manure GHG and ammonia (NH_3_) emissions. In a typical western Canadian beef feedlot, cattle are held in pens, and the dry solid manure is removed from the pens and spread onto nearby fields as a source of nutrients and organic matter^17^. Manure is often stockpiled or composted prior to application to fields^17,18^. Stockpiles heap manure into stacks with no active management of the pile^19^, while manure piles that are composted are turned or otherwise aerated^20^. Composting introduces oxygen (O_2_) into the manure piles, so it is generally expected that composted manure emits more aerobically-produced gases like carbon dioxide (CO_2_) and NH_3,_^19,21^ while stockpiled manure is expected to emit more anaerobically-produced gases like CH_4_^20^. Both high^18,22,23^ and low^24^ N_2_O emissions from composted manure have been reported. Emissions of these gases are related to manure carbon (C) and nitrogen (N) components, the O_2_ concentrations and other environmental conditions during decomposition.

No studies have assessed GHG and NH_3_ emissions from stored manure from cattle supplemented with 3-NOP. It is not known whether supplementing cattle with 3-NOP and monensin affects manure decomposition during storage. Mass balance estimates of manure C and N adjust concentrations to compensate for losses in dry matter during storage^17^. Total C and N mass balances could help evaluate whether processes during decomposition differ for manure from cattle supplemented with 3-NOP compared to manure from cattle that did not receive the supplement. If there are differences in manure C and N mass balances during storage because of the addition of 3-NOP and monensin to the diet, the resulting manure GHG and NH_3_ emissions may also be affected. Whether the GHG reductions achieved from lower enteric CH_4_ production with 3-NOP supplementation are negated by alterations to GHG and NH_3_ emissions during manure storage has not been assessed.

The objective of this research was to test if total carbon (TC) and total nitrogen (TN) mass balances, and GHG (CH_4_, CO_2_ and N_2_O) and NH_3_ emissions, from stockpiled and composted manure differed when manure piles were constructed with manure from cattle that received dietary 3-NOP or 3-NOP and monensin (3-NOP + Mon) compared to manure from cattle that were not fed these additives.

## Results

### Climate and pile characteristics

**Figure 1:**
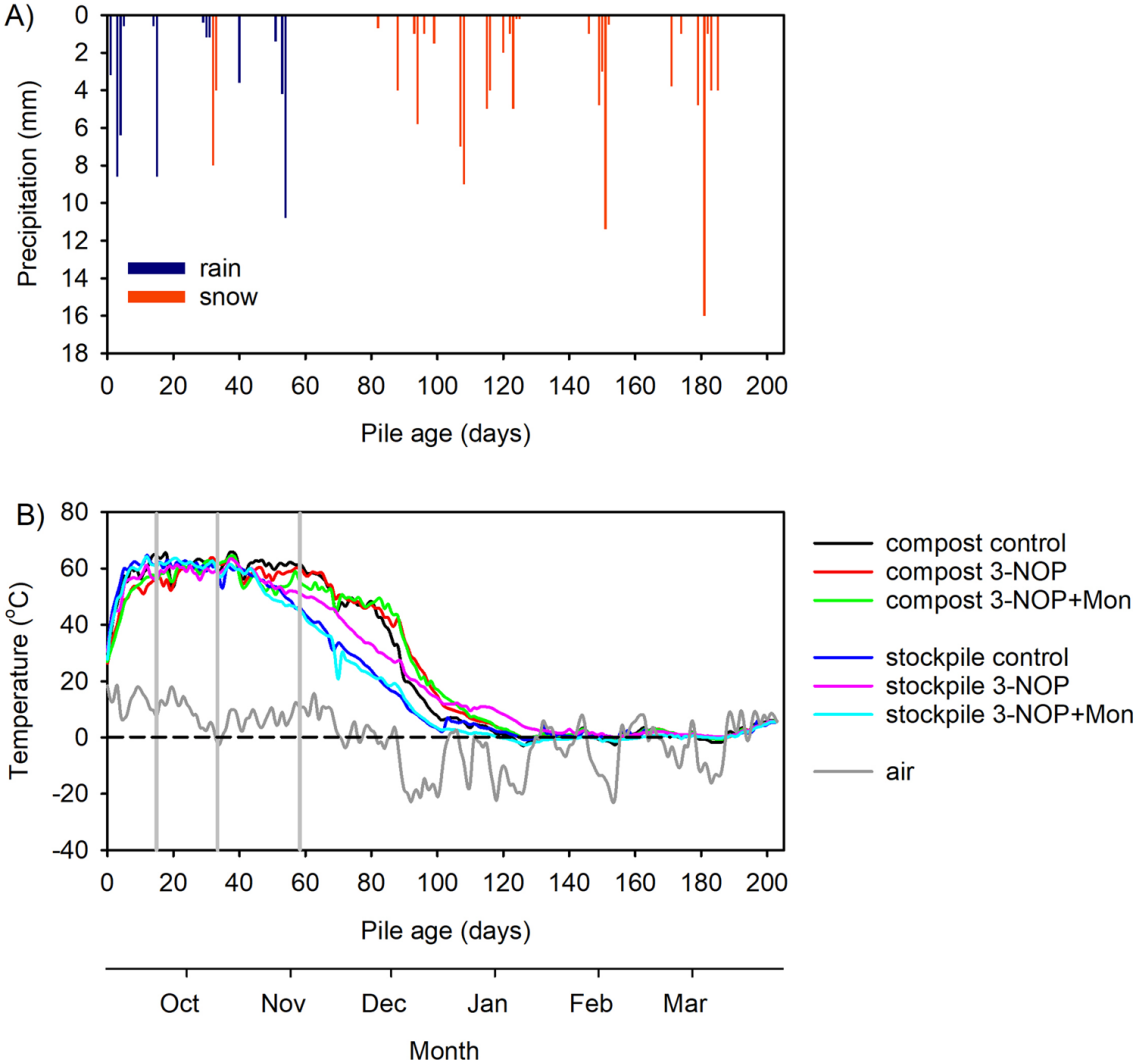
(**a**) Precipitation as rain and snow and (**b**) air temperature and mean pile temperatures by treatment (control, no supplements; 3-NOP, 3-nitrooxypropanol; 3-NOP + Mon, 3-nitrooxypropanol and monensin) for the stockpile and compost from September 2016 to March 2017.

During the 202-day trial (September 6, 2016, to March 28, 2017), there were 40 days of measurable precipitation (total = 116.8 mm, rain = 22.6 mm, snow = 116.8 cm, Fig. 1a). Daily average air temperature followed typical seasonal trends for the Canadian prairies from September to March, ranging from − 30 to 20 °C (Fig. 1b).

At the start of the trial, the manure was dry (0.34 kg kg^−1^). The composted manure surface area ranged from 36 to 38 m^2^ (average initial length = 6.72 m, width = 4.94 m and height = 1.37 m) and the stockpiled manure surface area averaged 26 m^2^ (average initial length = 5.31 m, width = 4.93 m and height = 1.32 m). The initial wet mass of the composted manure piles averaged 14,278 kg (± 292 kg s.d.) and the initial wet mass of the stockpiled manure piles averaged 9,665 kg (± 201 kg).

All manure piles reached internal temperatures of ~ 60 °C (Fig. 1b). Mean internal pile temperature between days 1 and 104 was 8 °C higher in the composted manure than in the stockpiled manure. Between days 1 and 104, the average pile temperature of the composted manure was 47 °C higher and stockpiled manure was 39 °C higher than between days 105 and 202 as decomposition rates, and therefore internal pile temperature, decreased after day 105 (Fig. 1b).

### Gas emissions

#### Carbon dioxide

**Figure 2:**
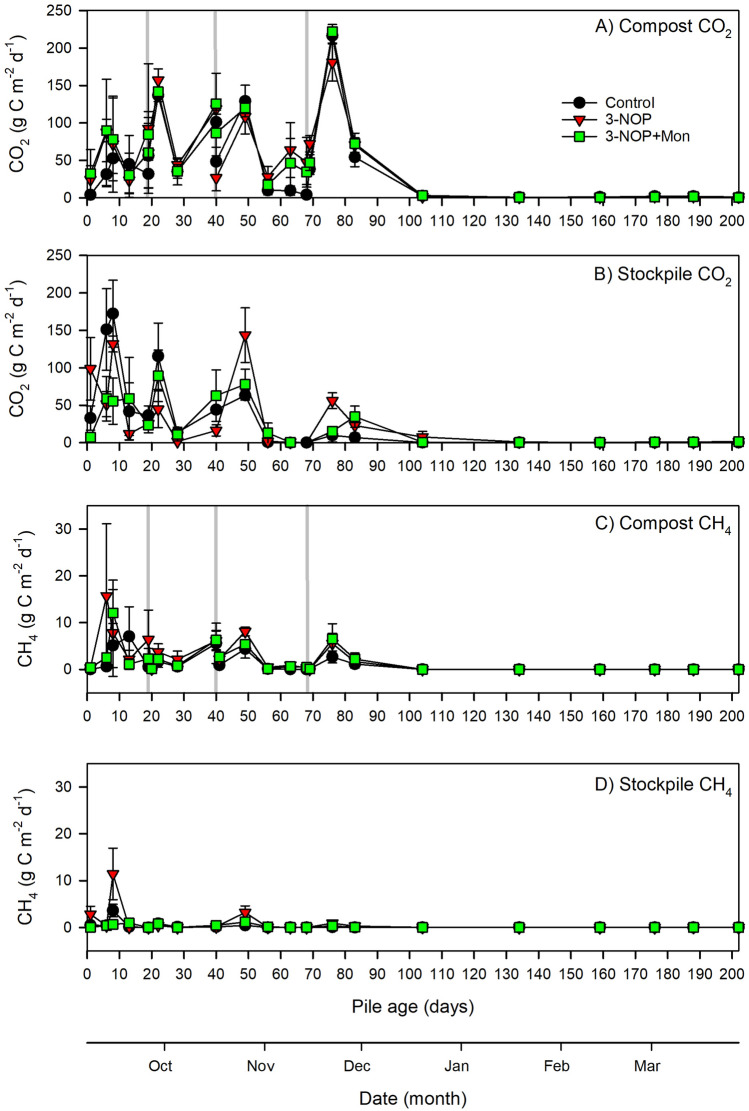
Daily surface-to-atmosphere carbon dioxide fluxes from the (**a**) composted and (**b**) stockpiled manure and methane fluxes from the (**c**) composted and (**d**) stockpiled manure from days 0 to 202. Treatment daily means (control is no supplements, 3-NOP is 3-nitrooxypropanol and 3-NOP + Mon is 3-nitrooxypropanol and monensin) are presented ± the standard error of the mean.

Between days 1 and 104, CO_2_ fluxes from the composted manure averaged 66.9 g C m^−2^ d^−1^ and from the stockpiled manure averaged 40.3 g C m^−2^ d^−1^ (Fig. 2a, b). Carbon dioxide fluxes were lower between days 105 and 202, averaging 1.2 g C m^−2^ d^−1^ from the composted manure and 0.7 g C m^−2^ d^−1^ from the stockpiled manure.

Between days 1 and 104, CO_2_ flux trends differed by handling method over time. Stockpiled manure CO_2_ fluxes were high at the beginning of the trial and generally declined with time (Fig. 2a). Composted manure CO_2_ fluxes were low at the start of the trial and increased after compost turning (Fig. 2b). The control, 3-NOP and 3-NOP + Mon treatments showed similar patterns of variability over time within the same handling method with some daily between-treatment variation noted within the first 20 days.

Cumulative CO_2_ emissions did not significantly differ by additive treatment, but they were affected by handling method when averaged across all additive treatments (Table 1). Compared to stockpiled manure, cumulative CO_2_ emissions from composted manure were on average 102% higher when expressed as kg C m^−2^ (*P* < 0.001), 179% higher when expressed as kg C pile^−1^ (pile surface area, *P* < 0.001) and 162% higher when expressed as CO_2_ kg C tonne^−1^ of initial C (*P* < 0.001).

**Table 1:** Cumulative gas emissions over the entire trial by additive treatment and pile handling method.

Units	Treatment	Handling method					
		Compost	Stockpile		Compost	Stockpile	
		CO_2_			CH_4_		
kg C m^−2^	All	6.37 (0.35)	3.15 (0.34)	***	0.23 (0.05)	0.04 (0.01)	***
	Control	5.65 (0.52)	3.10 (1.00)		0.17 (0.10)	0.02 (0.01)	
	3-NOP	6.60 (0.15)	3.27 (0.31)		0.30 (0.09)	0.08 (0.03)	
	3-NOP + Mon	6.86 (0.89)	3.08 (0.53)		0.23 (0.07)	0.03 (0.01)	
kg C pile^−1^	All	231.3 (14.35)	82.89 (8.81)	***	8.32 (1.69)	1.18 (0.33)	**
	Control	209.73 (10.22)	82.07 (26.6)		5.91 (3.41)	0.63 (0.20)	
	3-NOP	224.38 (3.61)	86.09 (5.92)		10.14 (2.98)	2.16 (0.71)	
	3-NOP + Mon	259.78 (41.13)	80.52 (13.43)		8.91 (2.99)	0.75 (0.25)	
kg C tonne^−1^ TC	All	168.23 (14.14)	64.22 (6.80)	***	5.97 (1.23)	0.94 (0.28)	***
	Control	155.27 (2.61)	62.93 (20.61)		4.26 (2.34)	0.48 (0.16)	
	3-NOP	149.94 (1.93)	67.55 (3.11)		6.72 (1.90)	1.74 (0.62)	
	3-NOP + Mon	199.49 (40.62)	62.17 (10.59)		6.93 (2.63)	0.59 (0.21)	

		N_2_O			NH_3_		
kg N m^−2^	All	0.04 (0.01)	0.04 (0.01)		0.22 (0.02)	0.12 (0.02)	***
	Control	0.03 (0.00)	0.03 (0.01)		0.26 (0.05)	0.14 (0.01)	
	3-NOP	0.05 (0.01)	0.05 (0.01)		0.20 (0.02)	0.10 (0.04)	
	3-NOP + Mon	0.04 (0.01)	0.05 (0.01)		0.20 (0.05)	0.11 (0.04)	
kg N pile^−1^	All	1.39 (0.19)	1.18 (0.16)		8.10 (0.92)	3.14 (0.48)	***
	Control	1.04 (0.17)	0.93 (0.35)		9.95 (2.18)	3.80 (0.06)	
	3-NOP	1.62 (0.23)	1.23 (0.21)		6.86 (0.54)	2.66 (1.06)	
	3-NOP + Mon	1.51 (0.51)	1.37 (0.31)		7.51 (1.60)	2.95 (1.16)	
kg N tonne^−1^ TN	All	0.080 (0.012)	0.072 (0.010)		0.46 (0.06)	0.19 (0.03)	***
	Control	0.061 (0.010)	0.056 (0.021)		0.59 (0.14)	0.23 (0.00)	
	3-NOP	0.085 (0.013)	0.076 (0.012)		0.36 (0.02)	0.17 (0.07)	
	3-NOP + Mon	0.093 (0.035)	0.084 (0.021)		0.44 (0.09)	0.18 (0.07)	

Source of variation	*P* value						
	CO_2_	CH_4_	N_2_O	NH_3_			
**kg N or C m**^**−2**^							
P	< 0.001	< 0.001	0.353	< 0.001			
T	0.406	0.355	0.139	0.101			
P × T	0.457	0.813	0.711	0.826			
**kg N or C pile**^**−1**^							
P	< 0.001	0.003	0.356	< 0.001			
T	0.397	0.464	0.213	1.03			
P × T	0.326	0.776	0.855	0.552			
**kg tonne**^**−1**^** TN or TC**							
P	< 0.001	< 0.001	0.613	< 0.001			
T	0.337	0.528	0.275	0.101			
P × T	0.245	0.746	0.989	0.413			

The means are presented with ± standard error of the mean in parentheses for each additive treatment (control, no supplements; 3-NOP, 3-nitrooxypropanol; 3-NOP + Mon, 3-nitrooxypropanol and monensin, n = 3). Asterisks in the “All” columns *,**,*** indicate significant differences between handling methods (averaged over all additive treatments) at *P* < 0.05, *P* < 0.01, and *P* < 0.001, respectively. The *P* values are for the mixed effects ANOVA model results for pile handling method (P), additive treatment (T) and their interactions.

#### Methane

Between days 1 and 104, CH_4_ fluxes averaged 2.6 g C m^−2^ d^−1^ from the composted manure and 0.7 g C m^−2^ d^−1^ from the stockpiled manure (Fig. 2c, d). Methane fluxes were negligible between days 105 and 202, averaging 7.5 × 10^−4^ g m^−2^ d^−1^ from the composted manure and 8.5 × 10^−5^ g C m^−2^ d^−1^ from the stockpiled manure.

Daily CH_4_ fluxes varied throughout the trial from the composted manure, with maximum fluxes occurring within one to two weeks after pile construction for all additives (Fig. 2c). While the timing of the maximum peaks in CH_4_ fluxes varied among additives, the temporal trends in CH_4_ fluxes from the composted manure were similar from all additives after the first compost turning on day 19. Generally, stockpiled manure CH_4_ emissions were low, with small spikes occurring at days 1, 8 and 49 of the trial (Fig. 2d). On those days, the largest CH_4_ spikes occurred from the 3-NOP treatment.

When averaged over all additives, cumulative CH_4_ emissions were 5.8 fold higher from the composted manure compared to the stockpiled manure when expressed as kg C m^−2^ (Table 1, *P* < 0.001). When expressed as CH_4_ kg C pile^−1^, cumulative CH_4_ emissions were 7.1 fold higher from the composted manure than from the stockpiled manure (*P* = 0.003). The CH_4_ kg C tonne^−1^ of initial C from the composted manure were 6.4 fold higher than from the stockpiled manure (*P* < 0.001).

#### Nitrous oxide

**Figure 3:**
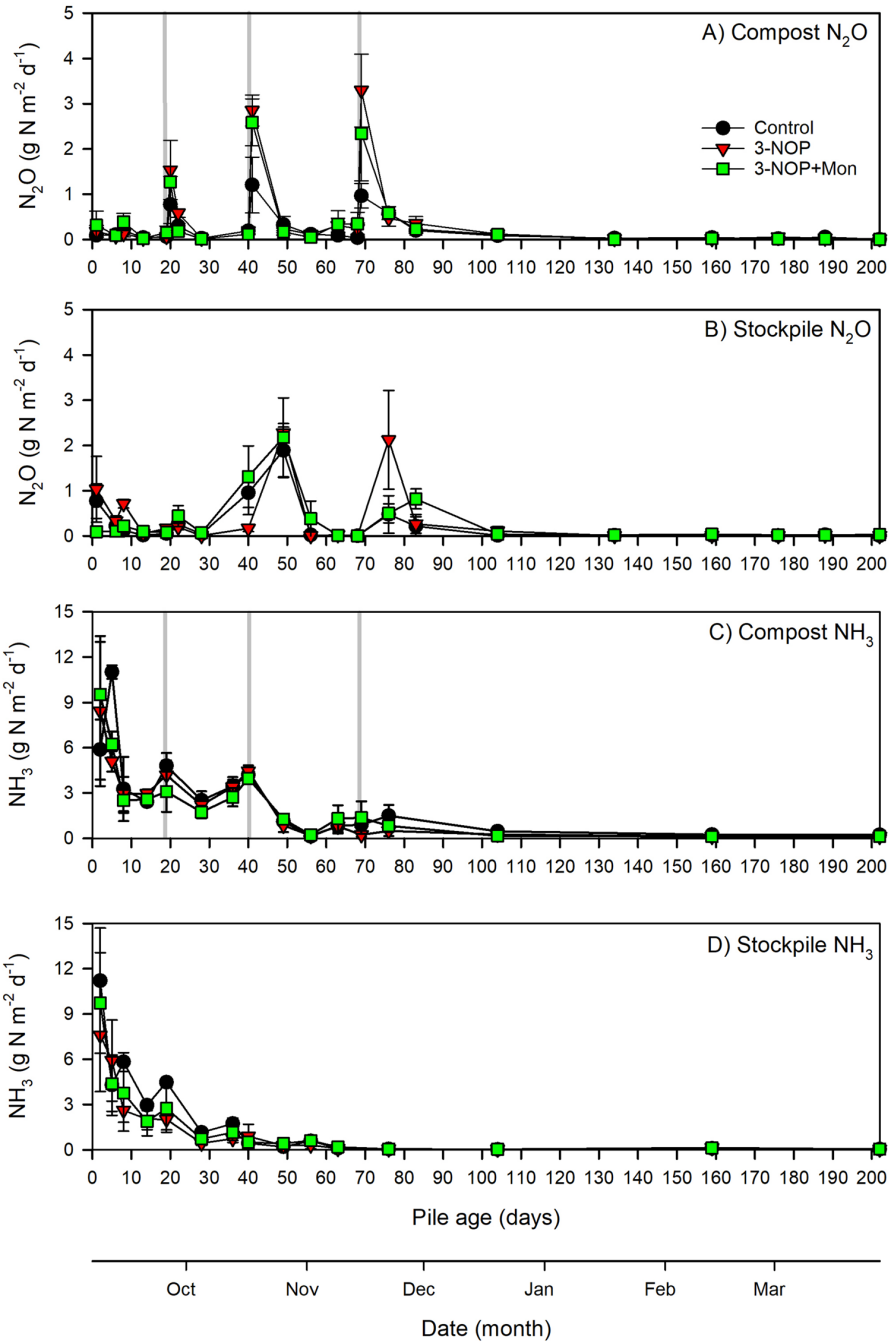
Daily surface-to-atmosphere nitrous oxide fluxes from the (**a**) composted and (**b**) stockpiled manure and ammonia fluxes from the (**c**) composted and (**d**) stockpiled manure from days 0 to 202. Treatment daily means (control in no supplements, 3-NOP is 3-nitrooxypropanol and 3-NOP + Mon is 3-nitrooxypropanol and monensin) are presented ± the standard error of the mean.

Between days 1 and 104, N_2_O fluxes averaged 0.5 g N m^−2^ d^−1^ from the composted manure and 0.4 g N m^−2^ d^−1^ from the stockpiled manure (Fig. 3a, b). From day 105 to 202, N_2_O fluxes averaged 0.03 g m^−2^ d^−1^ from the composted manure and 0.02 g m^−2^ d^−1^ from the stockpiled manure.

Daily N_2_O fluxes from composted manure were low throughout the trial. The exceptions were the measurements taken after compost turning where N_2_O fluxes from all additive treatments were between 6.2- to 24.8-fold higher when compared to measurements made the day prior to turning (Fig. 3a). From the stockpiled manure, daily N_2_O fluxes increased in all additives after day 28, but there were some differences in flux trends over time between additives: N_2_O fluxes from stockpiled 3-NOP manure were generally lower than the other additive treatments on day 40, and higher than other additive treatments on day 76 (Fig. 3b).

Despite some temporal discrepancies in daily N_2_O fluxes (Fig. 3a, b), there were no differences in cumulative N_2_O emitted from different handling methods, additive treatments, or any handling method by additive treatment interactions detected when emissions were presented as kg N m^−2^, kg N pile^−1^ or the kg N tonne^−1^ initial N (Table 1).

#### Ammonia

Between days 1 and 104, NH_3_ fluxes averaged 2.9 g N m^−2^ d^−1^ and 2.2 g N m^−2^ d^−1^ from the composted and stockpiled manure, respectively (Fig. 3c, d). Between days 105 and 202, NH_3_ fluxes averaged 0.1 g N m^−2^ d^−1^ from both composted and stockpiled manure.

Daily NH_3_ fluxes from composted and stockpiled manure were high early in the trial, peaking within the first six days after pile construction in all additive treatments (Fig. 3c, d). Within the respective handling methods, daily NH_3_ fluxes from all three additives were similar in magnitude throughout most of the trial and followed similar undulating trends.

There were no significant differences in cumulative NH_3_ emissions between the dietary additive treatments (Table 1). Cumulative NH_3_ emissions averaged over all additives on a kg m^−2^ basis were 83% higher from the composted compared to the stockpiled manure (*P* < 0.001). Cumulative NH_3_ emissions extrapolated over the initial surface area of the piles (kg pile ^−1^) averaged over all additives were 158% higher from the composted compared to the stockpiled manure (*P* < 0.001). Compared to the stockpiled manure, the NH_3_ kg N tonne^−1^ initial N was 142% higher (*P* < 0.001) from the composted manure.

### Manure chemistry

When averaged over all storage methods and additive treatments, manure TC concentrations, TN concentrations, C:N ratio, inorganic N concentrations, pH and electrical conductivity (EC) differed between days 1 and 202 (Table 2). All measured variables were higher on day 1 than on day 202 except pH and EC, which were higher on day 202. Manure storage affected TC concentrations; stockpiled manure TC concentrations at the end of the trial were lower (*P* < 0.10) than in initial samples, and composted manure TC concentrations at the end of the trial were lower (*P* < 0.10) than both the initial composted manure concentrations and the final stockpiled manure concentrations.

**Table 2:** Effects of manure handling method and additive treatments on manure chemical properties presented as mean ± standard error of the mean in parentheses.

Day	Handling method	Additive treatment	Total carbon	Total nitrogen	Inorganic nitrogen	Carbon: nitrogen	pH	EC
			kg tonne^−1^	kg tonne^−1^	kg tonne^−1^			ds cm^−1^
1	Initial	All	188.66 (6.24) ^¶^A	14.83 (0.50) A	1.692 (0.067) A	12.74 (0.14) A	8.90 (0.03) A	2118.2 (89.5) A
		Control	185.58 (13.29) de	14.74 (1.16) bcde	1.668 (0.153)	12.67 (0.20) bc	8.95 (0.04)	2206.6 (238.6)
		3-NOP	173.89 (8.63) cde	14.00 (0.66) bcde	1.751 (0.067)	12.41 (0.16) b	8.92 (0.04)	2127.7 (76.2)
		3-NOP + Mon	206.52 (7.78) e	15.76 (0.65) ce	1.656 (0.124)	13.14 (0.31) c	8.83 (0.05)	2020.3 (114.7)
202	Compost	All	125.88 (4.77) ^¶^B	11.71 (0.36) B	0.143 (0.005) B	10.73 (0.20) B	9.34 (0.04) B	2536.5 (64.3) B
		Control	113.69 (5.06) a	10.64 (0.40) a	0.159 (0.006)	10.69 (0.27) a	9.45 (0.03)	2774.8 (109.2)
		3-NOP	131.40 (6.27) ab	12.02 (0.46) ab	0.131 (0.008)	10.96 (0.43) a	9.26 (0.08)	2260.8 (66.8)
		3-NOP + Mon	132.55 (11.36) ab	12.46 (0.84) abd	0.140 (0.010)	10.55 (0.33) a	9.31 (0.08)	2574.0 (84.2)
202	Stockpile	All	145.54 (3.86) ^¶^C	13.12 (0.33) B	0.135 (0.007) B	11.13 (0.21) B	9.31 (0.06) B	2557.7 (88.10) B
		Control	146.90 (8.46) abcd	13.38 (0.66) abcde	0.140 (0.009)	10.99 (0.32) a	9.23 (0.08)	2576.1 (168.9)
		3-NOP	153.23 (5.09) abcd	13.77 (0.31) abcde	0.131 (0.017)	11.16 (0.39) a	9.37 (0.10)	2657.3 (171.8)
		3-NOP + Mon	136.49 (5.48) abc	12.22 (0.58) abc	0.133 (0.012)	11.25 (0.40) a	9.32 (0.11)	2439.7 (121.3)
Both	Compost	All	157.27 (5.81)^†^A	13.27 (0.37)	0.917 (0.111)	11.74 (0.18)	9.12 (0.04)	2327.4 (61.7)
Both	Stockpile	All	167.10 (4.69) B	13.98 (0.32)	0.913 (0.112)	11.94 (0.17)	9.10 (0.04)	2337.9 (69.1)
Both	Both	Control	157.94 (7.18)^‡^A	13.38 (0.52)	0.909 (0.138)	11.75 (0.20)	9.14 (0.04)	2441.0 (102.6)
		3-NOP	158.10 (4.59) A	13.45 (0.29)	0.941 (0.139)	11.73 (0.19)	9.12 (0.05)	2293.4 (62.9)
		3-NOP + Mon	170.52 (7.29) B	14.05 (0.44)	0.896 (0.135)	12.02 (0.25)	9.07 (0.05)	2263.6 (67.1)

		Source of variation			*P* value			
		P	0.076	0.113	0.933	0.454	0.695	0.920
		T	0.077	0.359	0.745	0.212	0.253	0.148
		P × T	0.504	0.339	0.997	0.758	0.157	0.241
		D	< 0.001	< 0.001	< 0.001	< 0.001	< 0.001	< 0.001
		P × D	0.059	0.095	0.933	0.181	0.633	0.893
		T × D	0.007	0.075	0.626	0.055	0.451	0.678
		P × T × D	0.504	0.339	0.997	0.758	0.157	0.241

“All” represents an average of all additive treatments (n = 27). Treatments were: control, no supplements; 3-NOP, 3-nitrooxypropanol; 3-NOP + Mon, 3-nitrooxypropanol and monensin (n = 9). Day 1 samples were collected during pile construction, and day 202 samples were collected at the end of the trial from the composted and stockpiled manure. The *P* values are for the mixed effects ANOVA model results for pile handling method (P), additive treatment (T), day (D) and their interactions. Differences in capital letters represent differences between handling methods, days, and/or handling method by day interactions at *P* < 0.10. Differences in lower case letters represent differences between additive treatments, and additive treatment by day interactions at *P* < 0.10.

^¶^Capital letters compare handling method and day interactions.

^†^Capital letters compare handling methods.

^‡^Capital letters compare additive treatments.

Manure TC concentrations were significantly affected by the additive treatment (Table 2). When averaged over the entire trial, TC in the control (mean = 157.9 g C kg^−1^) and 3-NOP (mean = 158.1 g C kg^−1^) were lower (*P* ≤ 0.05) than in 3-NOP + Mon (mean = 170.5 g C kg^−1^).

Concentrations of TN were affected by day, decreasing between days 1 and 202 (*P* ≤ 0.10) in both the composted and stockpiled manure (Table 2), but TN was not affected by additive treatment or manure handling (Table 2).

Manure TC, TN and C:N ratios from the additive treatments were different between the beginning and end of the trial (Table 2). The TN, TC and C:N ratio for all additive treatments were lower on day 202 than on day 1 (*P* ≤ 0.10). The TN and C:N ratio for 3-NOP + Mon were higher than 3-NOP (*P* ≤ 0.10) on day 1, but by day 202, there were no differences between the additive treatments.

### Stored manure carbon and nitrogen mass balance estimates

The mass balance estimates considered the changes in concentrations to compensate for losses in mass from the manure piles during the trial. Mass losses of DM as well as TC and TN tended to be higher in composted compared to stockpiled manure, however, these differences were not significant (Table 3).

**Table 3:** Mean ± standard error of the mean in parentheses showing the percent losses of dry matter, total carbon and total nitrogen from each pile handling method (P) and additive treatment (T) between the start and end of the trial (n = 18) determined by the mass balance analysis.

Handling method	Additive treatment	Initial	Final	Total loss	Total loss
		kg tonne^−1^	kg tonne^−1^	kg tonne^−1^	%
Dry matter					
Compost	All	1000	850.9 (26.1)	149.1 (26.1)	15.8 (2.6)
	Control	1000	831.3 (34.9)	168.7 (34.9)	16.9 (3.4)
	3-NOP	1000	896.3 (8.6)	103.7 (8.6)	11.3 (0.8)
	3-NOP + Mon	1000	825.1 (73.0)	174.9 (73.0)	19.1 (7.1)
Stockpile	All	1000	891.8 (22.7)	108.2 (22.7)	13.9 (2.3)
	Control	1000	903.0 (41.7)	97.0 (41.7)	12.0 (4.4)
	3-NOP	1000	946.3 (9.2)	53.7 (9.2)	8.8 (0.8)
	3-NOP + Mon	1000	826.0 (25.8)	174.0 (25.8)	21.1 (2.5)
Both	Control	1000	867.2 (29.1)	132.8 (29.1)	14.5 (2.7) ab
	3-NOP	1000	921.3 (12.5)	78.7 (12.5)	10.1 (0.8) a
	3-NOP + Mon	1000	825.5 (34.6)	174.5 (34.6)	20.1 (3.4) b

Source of variation					*P* value
	P				0.228
	T				0.093
	P × T				0.661

Total carbon					
Compost	All	188.7 (7.5)	108.3 (9.4)	80.4 (14.1)	40.6 (5.8)
	Control	185.6 (13.5)	94.6 (5.7)	90.9 (18.8)	45.4 (6.2)
	3-NOP	173.9 (6.7)	118.0 (11.3)	55.9 (4.6)	31.7 (2.6)
	3-NOP + Mon	206.5 (13.1)	112.2 (27.6)	94.3 (39.4)	44.5 (17.1)
Stockpile	All	188.7 (7.5)	130.3 (7.1)	58.3 (12.3)	29.9 (6.1)
	Control	185.6 (13.5)	133.3 (16.5)	52.3 (22.5)	24.7 (13.3)
	3-NOP	173.9 (6.7)	144.9 (1.8)	28.9 (4.9)	18.0 (3.3)
	3-NOP + Mon	206.5 (13.1)	112.7 (7.8)	93.8 (13.9)	47.2 (4.7)
Both	Control	185.6 (8.6)	114.0 (11.6)	71.6 (15.7)	35.1 (8.0)
	3-NOP	173.9 (4.3)	131.5 (7.9)	42.4 (6.8)	24.9 (3.6)
	3-NOP + Mon	206.5 (8.3)	112.5 (12.8)	94.1 (18.7)	45.9 (8.0)

Source of variation					*P* value
	P				0.146
	T				0.136
	P × T				0.472

Total nitrogen					
Compost	All	14.8 (0.5)	10.0 (0.8)	4.8 (1.1)	37.3 (4.4)
	Control	14.7 (1.3)	8.8 (0.2)	5.9 (1.5)	40.2 (6.9)
	3-NOP	14.0 (0.3)	10.8 (0.6)	3.2 (0.5)	26.5 (6.5)
	3-NOP + Mon	15.8 (1.0)	10.5 (2.3)	5.3 (3.0)	49.2 (2.6)
Stockpile	All	14.8 (0.5)	11.8 (0.7)	3.1 (0.9)	28.4 (3.5)
	Control	14.7 (1.3)	12.1 (1.4)	2.6 (1.9)	33.5 (3.4)
	3-NOP	14.0 (0.3)	13.0 (0.3)	0.9 (0.5)	18.6 (6.3)
	3-NOP + Mon	15.8 (1.0)	10.1 (0.9)	5.6 (1.1)	33.0 (4.4)
Both	Control	14.7 (0.8)	10.5 (1.0)	4.3 (1.3)	36.9 (3.7)
	3-NOP	14.0 (0.2)	11.9 (0.6)	2.1 (0.6)	22.6 (4.4)
	3-NOP + Mon	15.8 (0.6)	10.3 (1.1)	5.4 (1.4)	39.5 (4.3)

Source of variation					*P* value
	P				0.178
	T				0.197
	P × T				0.401

The *P* values for the mixed effects ANOVA model results for P, T and P and T interactions for total % losses. Differences in lower case letters represent differences between treatments at *P* < 0.10.

From the composted manure, the mass balance showed an equivalent of 90.9 kg C tonne^−1^ was lost from the control and 94.3 kg C tonne^−1^ was lost from 3-NOP + Mon, while only 55.9 kg C tonne^−1^ was lost from 3-NOP. Likewise, from the composted manure, an equivalent of 5.9 kg N tonne^−1^ was lost from the control and 5.3 kg N tonne^−1^ was lost from 3-NOP + Mon, while 3.2 kg N tonne^−1^ was lost from 3-NOP. While 3-NOP showed slightly lower mass losses of TC and TN compared to the control and 3-NOP + Mon in the composted manure, due to the high variability, these differences were not statistically significant (Table 3). The combination of slightly greater TC and TN losses from 3-NOP resulted in greater (*P* < 0.10) DM losses in 3-NOP compared to 3-NOP+Mon (Table 3).

There were discrepancies between the losses calculated using the mass balance approach (Table 3) and the GHGs lost as a fraction of the initial TC and TN in the compost (Table 1). It appears that more TC was lost as CO_2_ and CH_4_ than were lost as a fraction of the initial TC when calculated using the mass balance approach. The mass losses of TN were higher than the cumulative losses of N as NH_3_ and N_2_O (Table 1).

Across all additive treatments in the stockpiled manure, the mass balance calculations estimated that total losses of C averaged 58.3 kg C tonne^−1^ and total losses of N averaged 3.1 kg N tonne^−1^ (Table 3). The total losses of C estimated using the mass balance approach from the stockpiled manure were similar to the cumulative losses of CO_2_ and CH_4_ expressed as kg C tonne^−1^ initial C from the stockpiled manure (equating to 65.2 kg C tonne^−1^ C). The TN losses calculated using the mass balance approach (Table 3) were higher than those determined by cumulative losses of N_2_O or NH_3_ as kg N tonne^−1^ initial N from the stockpiled manure (Table 1).

## Discussion

This was the first study to assess whether GHG and NH_3_ emissions from composted and stockpiled beef cattle manure are affected by dietary supplementation with 3-NOP or a combination of 3-NOP and monensin. While 3-NOP and monensin supplementation can reduce enteric CH_4_ production from ruminant animals^10,13,14^, we found that they did not significantly affect cumulative GHGs or NH_3_ emissions from stored manure. Stored manure TC concentrations were affected by these dietary additives. However, the additives did not significantly alter mass balance estimates of TC and TN in composted or stockpiled manure.

In our study, a combination of 3-NOP and monensin resulted in significantly higher manure TC concentrations compared to the control and 3-NOP. This suggests there could be differences in the relative amounts of recalcitrant versus labile forms of C in manure from cattle supplemented with 3-NOP and monensin. A previous study noted 3-NOP-induced increases in excreted neutral (hemicellulose, cellulose and lignin) and acid detergent fibre (cellulose and lignin) in fresh manure from lactating dairy cows^14^, and monensin has been shown to alter digestibility of NDF and ADF in cattle^10^. Future studies should consider using more sophisticated methods to gather insights into these differences. When mass losses were considered, we did not find significant differences in TC and TN between the additive treatments suggesting that microbial activity during manure decomposition was not significantly affected by the additive treatments.

This was the first study to evaluate the potential impacts of 3-NOP on GHG and NH_3_ emissions from stored manure. Therefore, there are no directly comparable data sets. Additive treatments did not significantly affect cumulative GHGs or NH_3_ emissions. This is consistent with the lack of mass balance differences in TC and TN for stored manure between additive treatments. For GHG or NH_3_ emissions to be affected by additives, differences in substrates or microbial communities and/or microbial activity within the manure pile would be required. Since changes in stored manure TC and TN are largely the result of microbial activity, there is no evidence that 3-NOP had a significant effect on these factors in our study.

It was expected that there would be differences between the composted and stockpiled manure. There was slightly greater thermophilic decomposition in the composted manure compared to the stockpiled manure as indicated by a longer duration of relatively higher internal pile temperatures^25,26^. However, we did not observe differences in mass losses of DM, TC or TN as a result of the different storage methods (Table 3). This is in contrast to the results of other studies which have noted greater losses of TC and TN from composted manure compared to stockpiled manure^17^. Our results may be a consequence of low manure moisture content initially and throughout which were less-than-optimal for manure composting^27^. They may also be attributed to the low compost turning frequency (three times) compared to the six to eight times in other studies as greater turning frequency leads to greater decomposition and therefore greater DM, TC and TN losses^28^.

Trends in daily fluxes observed during our trial were similar to those observed during other studies. Similar to our results, others have also reported CH_4_ emissions from composted and stockpiled cattle manure spiked shortly after pile creation^18,29^ and that CO_2_ emissions from composted and stockpiled beef cattle manure were initially high and decreased over time^18,24^. Composting manure has been shown to result in high variability of N_2_O emissions^18,24^, and others have similarly reported N_2_O fluxes increased after NH_3_ fluxes decreased^24,30^.

Composting manure resulted in higher cumulative CO_2_ and NH_3_ emissions compared to stockpiled manure which is consistent with other studies^22,28^. Compost turning increases aeration and aerobic decomposition of organic material and hydrolysis of organic N resulting in the production of CO_2_ and NH_3_^24,26^. Because turning introduces O_2_ into the compost, it was a little surprising that we observed higher cumulative CH_4_ emissions from the composted manure compared to the stockpiled manure.

Methane production requires the absence of O_2_ as a precondition for production^31^ and stockpiled manure can have high CH_4_ emissions^20^. However, anaerobic conditions do not always form in stockpiled manure^18^ and high CH_4_ production within manure piles does not always equate to greater surface-to-atmosphere CH_4_ emissions^23^. In the current study, low CH_4_ emissions from our stockpiled manure can be partially attributed to the initial pile characteristics as the manure was mixed with straw bedding and the initial moisture content was ~ 0.34 kg kg^−1^ which is lower than the > 0.60 kg kg^−1^ initial manure moisture values reported in other studies that measured high CH_4_ emissions from stockpiled manure^22,32,33^. These characteristics can contribute to low density and high porosity in the manure piles, which allows diffusion of O_2_ into the pile from the atmosphere. This may have allowed aerobic conditions to predominate inside the stockpiled manure resulting in low CH_4_ emissions^34^. Likewise in these conditions, CH_4_ produced within the pile may be oxidized before being emitted to the atmosphere^23^. The lower cumulative CH_4_ emissions from the stockpiled compared to the composted manure may also be attributed to the relatively smaller pile size of the stockpiles as CH_4_ emissions have been found to be lower when manure piles are smaller^30^.

We found no differences in cumulative N_2_O emissions between the composted manure and stockpiled manure. There was some evidence that reducing conditions were developing within the stockpiled manure after day 30 as shown by the increase in N_2_O fluxes, while in the composted manure most of the N_2_O was emitted after turning. During manure storage, nitrification on the pile surface leads to accumulation of NO_2_^-^ and NO_3_^-^ and these substrates are reintroduced into the compost pile during turning contributing to spikes in N_2_O fluxes from denitrification^23,26^. The data suggests the environmental conditions and processes occurring in the composted and stockpiled manure differed, however, they emitted similar amounts of N_2_O.

The mass balance estimates suggest more N was lost from both the composted and stockpiled manure than was accounted for as NH_3_ or N_2_O. Some of the discrepancies could be related to gases that we have not accounted for (i.e. nitric oxide and nitrogen gas)^35^. The losses of TC from the stockpiled manure estimated by the mass balance (All = 58.3 kg tonne^−1^) were similar to the total losses of CO_2_ + CH_4_ kg C tonne^−1^ TC (All = 65.1 kg C tonne^−1^). However, mass balance estimates of TC losses (All = 80.4 kg tonne^−1^) and the losses of CO_2_ + CH_4_ kg C tonne^−1^ TC (All = 174.3 kg C tonne^−1^) were not comparable in the composted manure. Previous studies have found similarities between these values^36^. The mass balance estimates use the initial and final manure samples and mass balances are derived from an empirical model^37^. This method has been shown to sometimes cause erroneous values when manure samples are not representative of the entire pile, or when soil is introduced into the compost piles during turning^38^. These factors may have impacted our results. Despite these discrepancies, both the gas and mass balance data consistently showed no significant effects on C or N cycling in stored manure piles resulting from supplementing beef cattle with 3-NOP or 3-NOP and monensin. Future studies should consider placing manure piles on concrete pads to prevent mixing soil with the manure, and collecting multiple samples at various areas of the manure piles to ensure samples are representative.

Measurement of GHGs using static chambers can contribute to errors. There are inaccuracies associated with extrapolating 40 min static chamber measurements made one to three times a week over the entire 202 day trial. Temporal extrapolation of GHG fluxes from static chambers are associated with some error in cumulative GHG emissions when used on soils^39^. Likewise, the area covered by the chambers was small so we only measured a fraction of the total surface area of the manure piles.

Our study did not evaluate the potential effects of differences in 3-NOP and monensin dosage, and we also did not consider differences in cattle diets. The effects of 3-NOP on the digestibility of organic matter in manure have been found to vary slightly by dose^16^ and enteric CH_4_ production has been found to be related to diet^40^. Thus our results might not be replicated under different 3-NOP doses and different diets.

The presence of residual monensin in the manure was not considered during our study. Monensin in its active form can be excreted with manure, and supplementation with monensin could alter GHG emissions from manure^11,41^. However, both composting and stockpiling manure have been shown to reduce monensin concentrations in stored manure^42^. Future studies should consider incorporating extractions to test for monensin over time during manure storage to properly evaluate whether GHG emissions from stored manure are directly affected by monensin.

Management activities such as adding water to the manure piles, swapping straw for other materials^29,32^, changing cattle diet^43^ and changing compost turning frequency^28^ affect the degradation of residual dietary additives in manure, as well as manure components and GHG and NH_3_ emissions. These factors have not been considered in the current study.

In summary, our study found some differences in manure chemistry as well as higher CO_2_, CH_4_ and NH_3_ emissions from composted compared to stockpiled manure, which can be attributed to differences in conditions resulting from differences in handling strategies and pile characteristics. We found that supplementing cattle with 3-NOP and monensin affected the initial manure TC concentrations, but they did not significantly influence estimates of TC or TN mass balances in stored manure. As a result, there were no significant differences between additive treatments for cumulative GHGs and NH_3_ emissions from composted and stockpiled manure. The lack of differences between additive treatments in our trial suggests that manure from cattle supplemented 3-NOP does not require manure storage that differs from current strategies. Our trial results provide assurance that enteric CH_4_ reductions achieved through dietary supplementation with 3-NOP are not negated by increases in GHG emissions during manure storage. This study can serve as a reference for policy makers that will want to know about any confounding effects that supplements have on GHG emissions and nutrient transformations in manure from beef cattle feedlots.

## Methods

This experiment was conducted in semiarid Lethbridge, Alberta, Canada (49°42′03.3"N, 112°45′51.0"W). Precipitation data was acquired from an Agriculture and Agri-Food Canada weather station located < 1 km from the trial.

### Manure collection

Manure mixed with cereal straw (manure:straw ratio of 4:1) was collected from a congruent feeding trial that was conducted from November 2015 to August 2016^44^. All cattle were fed the same high-forage diet. Steers were fed a backgrounding diet for 105 days consisting of 65% barley (*Hordem vulgare*) silage, 5% supplement (vitamins, minerals and crude protein), and 30% dry rolled barley and then were transitioned to a finishing diet over 28 days. The finishing diet consisted of 8% barley silage, 5% supplements, and 87% dry rolled barley grain for 105 days. A full profile of feed ingredients and chemical composition of dietary ingredients are presented in Vyas et al.^44^.

In separate pens, three treatments were established based on the addition or exclusion of additives. The control group did not receive additional supplements, the “3-NOP” group received 3-NOP, and the “3-NOP + Mon” group were supplemented with 3-NOP and monensin. During backgrounding, 3-NOP was supplemented at 200 mg kg^−1^ dry matter (DM) and monensin was supplemented at 33 mg kg^−1^ DM. During finishing, 3-NOP was supplemented at 125 mg kg^−1^ DM and monensin was supplemented at 33 mg kg^−1^ DM.

### Experimental setup

Manure was removed from the feedlot on September 6, 2016, at the end of the associated feeding trial^44^. Manure from each treatment was separately transported from the feedlot to the experimental site. Each pile was separated by a buffer of 3.65 m in the east–west direction and 7.62 m in the north–south direction. The piles were divided into two blocks by handling method: composting and stockpiling. Three replicates of each handling method (composting and stockpiling) and treatment (control, 3-NOP and 3-NOP + Mon) were randomly distributed throughout their respective blocks for a total of 18 manure piles.

The initial masses of each compost pile and stockpile were determined during the pile creation from the manure spreader’s on-board scale. Dimensions of each pile were determined by manually measuring the length, width, circumference and height of piles with a tape measure. This data was used to determine pile surface area.

For the stockpiles, manure was heaped into piles roughly conical in shape. For the composted manure, windrows were created. All manure piles were unconfined and situated on soil.

The piles were uncovered for the duration of the trial (202 days). The trial duration was dictated by the temperature, which decreases when manure decomposition slows. The manure remained stored until the spring—even after decomposition had slowed—when it could be applied to the soil. The compost piles were turned on September 26, 2016 (day 19), October 17, 2016 (day 40) and November 15, 2016 (day 69) using an Earthsaver windrow turner (Fuel Harvesters Equipment, Midland, TX, USA). The stockpiles were left undisturbed for the duration of the trial.

### Manure properties and analyses

Air and internal manure pile temperatures at the top, middle and bottom of the piles were measured hourly during the trial using T-wire thermocouples recorded by a datalogger (CR800 with an AM16/32B channel relay multiplexer, Campbell Scientific, Logan, UT, USA). The thermocouples were briefly removed from the compost piles during turning. Pile temperatures were averaged into one temperature representative of each treatment and handling method.

At the start and end of the trial, each manure pile was cut perpendicular to its length to expose a vertical face. Manure was sampled to determine manure properties from the top, middle and bottom of each pile. The values were averaged for each treatment and handling method.

Gravimetric moisture content was determined by drying 100 g of manure at 60 °C for one week. The moisture content was expressed on a wet basis while manure C and N concentrations were expressed on a dry basis.

Manure pH and EC (ds cm^−1^) were obtained by mixing 15 g of fresh sample and 150 mL ultrapure water for 30 min, then centrifuging (10,000 rpm for 10 min). The pH and EC of the supernatant were measured on a Orion Star A215 pH & EC meter (Thermo Fisher Scientific, Waltham, MA, USA). The sample was then filtered (0.45 µm) and the supernatant solution was analysed for soluble inorganic N (ammonium, NH_4_^+^; nitrite, NO_2_^-^; nitrate, NO_3_^-^) using ion chromatography (ICS-1000 Ion Chromatography System, Dionex, Sunnyvale, CA, USA).

Manure TC and TN (kg tonne^−1^ DM manure) were determined from freeze-dried ground (≤ 0.15 mm) manure and measured using a CN analyser (Carlo Erba Instruments, Rodano, Italy) and expressed on a dry weight basis.

### Mass balance estimates

Mass losses (%) of DM, TC and TN were estimated from measurements of the initial and final samples from all additive treatments and both manure storage practices using equations outlined in Larney et al.^17^. Briefly, assuming an initial mass of 1000 kg of fresh manure, and using initial and final moisture measurements, DM losses were derived by assuming all mass losses were organic material and estimated according to an empirically derived relationship^37^. The TC and TN losses were derived by calculating the percent difference in their initial and the final concentrations relative to the initial and final masses of the manure piles.

### Greenhouse gas and ammonia sampling

Ammonia and GHG fluxes were measured one to three times a week from September 7, 2016 (day 1) until November 29, 2016 (the first 83 days, the thermophilic phase when internal manure pile temperatures were > 45 °C) then three to six more times thereafter until March 28, 2017 (day 202). For each handling method and additive treatment, this resulted in a total of 14 NH_3_ measurements. For stockpiled manure, we measured GHGs 15 times for each additive treatment, and from composted manure, we measured GHGs 18 times for each additive treatment. The three additional measurements made from the composted manure occurred immediately after the compost was turned.

Ammonia fluxes were measured using cylindrical PVC vented chambers (chamber area = 0.019 m^2^) with bases inserted 3–5 cm into the manure.

The chambers were equipped with two 2.5 cm thick polyurethane foam disks cut fit-to-seal to the inner diameter of the PVC chambers and infused with 50 mL of phosphoric-glycerol solution to trap gaseous NH_3_. The “measurement disk” was situated above the pile surface to capture NH_3_ emitted off the manure, and a “scrubbing disk” was inserted between the measurement disk and the atmosphere to prevent contamination of the measurement disk. Each chamber was covered with a canopy to prevent precipitation from washing the acid solution from the disks.

For measurements, the foam disks were brought to the laboratory in sealed bags, weighed, and the amount of solution absorbed into each disk was determined using before and after deployment weights. Disk NH_3_ concentrations were extracted by saturating each disk with 100 mL of 0.5 M KCl solution for 30 min. Extractions were frozen at − 20 °C prior to analysis. The extracts were analysed using the indophenol blue method with a MultiSkan Go Microplate Spectrophotometer at 650 nm (Thermo Scientific, Waltham, MA, USA). Ammonia fluxes were calculated by dividing the extraction concentrations by the chamber area and deployment time.

Greenhouse gas samples were collected from vented static chambers (area = 0.07 m^2^, volume = 7 L). Chamber bases (inner diameter = 30 cm) were inserted 5 cm into the manure pile. The bases were briefly removed and reinstalled before and after compost turning.

For GHG measurements, chamber covers were clipped to the bases and samples taken at 0, 10, 20 and 40 min after covering. From each chamber, 11 mL of gas was extracted from the headspace using a polypropylene syringe and immediately injected into a pre-evacuated (− 1 atm) 5.8 mL Exetainer (Labco Ltd., Lampeter, United Kingdom). Gas concentrations were determined using a gas chromatograph equipped with electron capture, thermal conductivity and flame ionization detectors (Varian 3800, Varian Instruments, Palo Alto, CA, USA). The injector and column temperatures were kept at 55 °C. The carrier was P10 gas (10% methane, balance argon) for N_2_O and helium for CO_2_ and CH_4_. The channel was maintained at a static pressure of 150 kPa.

Greenhouse gas fluxes from the surface of manure piles to the atmosphere were calculated using air temperature, the ideal gas law, chamber area and volume, and the change in gas concentration over time for each chamber. The change in gas concentration over time was assessed using both quadratic (QR) and linear regression (LR). Unless the second derivative of the model was ≤ 0 according to the LINEST function in Microsoft Excel (version 2019), fluxes were calculated with QR^45,46^. The minimum detectable fluxes were determined for CO_2_, CH_4_ and N_2_O^47^. The minimum detectable fluxes for quadratic and linear fluxes were: ± 2215.7 and ± 641.5 µg C m^−2^ h^−1^, respectively, for CO_2_; ± 2.8 and ± 9.7 µg N m^−2^ h^−1^, respectively, for N_2_O; and ± 4.9 and ± 16.8 µg C m^−2^ h^−1^, respectively, for CH_4_. Fluxes below the minimum detectable flux were assigned a value of zero. Of the 387 fluxes measured, the QR method was used for 85, 81 and 55% of the CO_2_, N_2_O and CH_4_ fluxes, respectively, the LR method was used for 12, 19 and 44% of the CO_2_, N_2_O and CH_4_ fluxes, respectively, and 3, 1 and 1% of the CO_2_, N_2_O and CH_4_ fluxes were below detection, respectively.

Daily NH_3_ and GHG flux rates for the composted and stockpiled manures are presented by additive treatment as well as an average for all treatments together (“All”) to compare flux rates between storage methods. The daily gas emissions are expressed as fluxes per unit area (g m^−2^ d^−1^).

The cumulative emissions from each pile were determined by integrating (trapezoidal method) between daily measurements and summing the values, which were then averaged based on additive treatment and storage method.

The cumulative emissions for each treatment and storage method are presented on a per unit area basis (kg m^−2^), averaged over the initial surface area of the manure piles (kg pile^−1^), and determined as a proportion of the initial manure TC and TN content (kg C or N tonne^−1^).

The GHG and NH_3_ emissions were extrapolated from a per unit area to per surface area of the pile by multiplying the emissions (kg m^−2^) by the initial surface area of the piles (pile^1^ as m^2^).

The emissions as kg tonne^−1^ initial manure TC or TN were calculated by dividing the initial surface area of the piles (m^2^) by the initial surface area, multiplying the emissions by the kg tonne^−1^ initial DM manure, then multiplying by the fraction of initial TC or TN in the manure.

### Data handling and statistics

Manure chemical concentrations, % losses of DM, TC and TN from mass balance estimates, and cumulative GHG and NH_3_ emissions were subject to statistical analyses. Data were analysed using R Statistics (version 3.4.3). Normality of each dataset was tested using a Kolmogorov–Smirnov test and homogeneity of variance assessed using Levene's test. None of the data sets were transformed. To test for differences in manure chemical concentrations by handling method (compost and stockpile), additive (control, 3-NOP and 3-NOP + Mon) and day (1 vs 202), a three-factor mixed model ANOVA was conducted (*nlme* package in R), and when appropriate, post-hoc analysis was completed using a Tukey’s HSD Test (*lsmeans* package in R). To test for differences in cumulative GHG and NH_3_ emissions and % mass losses of DM, TC and TN by handling method (compost and stockpile) and additive (control, 3-NOP and 3-NOP + Mon), a two-factor mixed model ANOVA was used. In the mixed models, replicate was treated as a random effect and all other factors included in the model were treated as fixed effects. Significance was evaluated at *P* ≤ 0.10.
